# Aberrant Interhemispheric Functional Organization in Children with Dyskinetic Cerebral Palsy

**DOI:** 10.1155/2019/4362539

**Published:** 2019-03-18

**Authors:** Yun Qin, Bo Sun, Huali Zhang, Yanan Li, Tao Zhang, Cheng Luo, Chengyan Sun, Dezhong Yao

**Affiliations:** ^1^The Clinical Hospital of Chengdu Brain Science Institute, MOE Key Lab for Neuroinformation, High-Field Magnetic Resonance Brain Imaging Key Laboratory of Sichuan Province, University of Electronic Science and Technology of China, Chengdu 610054, China; ^2^Sichuan Rehabilitation Hospital, Chengdu, China

## Abstract

**Background:**

Hemispheric asymmetry is one fundamental principle of neuronal organization. Interhemispheric connectivity and lateralization of intrinsic networks in the resting-state brain demonstrate the interhemispheric functional organization and can be affected by disease processes. This study aims to investigate the interhemispheric organization in children with dyskinetic cerebral palsy (DCP) based on resting-state functional MRI (fMRI).

**Methods:**

24 children with DCP and 20 healthy children were included. Voxel-mirrored homotopic connectivity (VMHC) was calculated to detect the interhemispheric connectivity, and the lateralization of the resting-state networks was performed to examine the asymmetry of the intrinsic networks of brain.

**Results:**

Decreased interhemispheric connectivity was found at visual, motor, and motor-control related regions in children with DCP, while high cognitive related networks including the central executive network, the frontoparietal network, and the salience network represented decreased asymmetry in children with DCP. Abnormal VMHC in visual areas, as well as the altered lateralization in inferior parietal lobule and supplementary motor area, showed correlation with the gross motor function and activities of daily living in children with DCP.

**Conclusion:**

These findings indicate that the interhemispheric functional organization alteration exists in children with DCP, suggesting that abnormal interhemispheric interaction may be a pathophysiological mechanism of motor and cognitive dysfunction of CP.

## 1. Introduction

Hemispheric asymmetry is one of the fundamental principles of neuronal organization, with profound effects on sensory, motor, and cognitive processing [1, 2]. It plays a crucial role in integrating information across the whole brain and maintaining the independent processing of the hemispheres. Strong interhemispheric interaction of sensorimotor, as well as lateralized organization between hemispheres associated with higher cognitive functions, is fundamental to healthy brain development [3]. Interhemispheric functional organization facilitates motor control and neural plasticity, and abnormal interhemispheric interaction was found to disturb the motor performance in many motor-related diseases [4–6].

Cerebral palsy (CP) encompasses a range of motor and postural disorders due to nonprogressive disturbances in fetal or infant development. Among all children with CP, approximately one-third have hemiplegic cerebral palsy caused by asymmetric lesion on a developing brain [7]. Dyskinetic CP (DCP), dominated by dystonic and choreoathetoid CP, is the second most common CP subtype, which is characterized by abnormal patterns of posture or movement, accompanied by involuntary, recurring, and uncontrolled and occasionally stereotyped movements [8, 9]. Heterogeneous damage to the brain has been found in children with DCP. Qualitative MR studies have showed that DCP is mainly associated with basal ganglia and thalamic injury [10, 11]. In addition, studies applying diffusion tensor imaging have also reported decreased white matter (WM) volume and fractional anisotropy (FA) in DCP subjects [12, 13]. Moreover, children with CP commonly experience impairments across multiple executive function domains with considerable impact on everyday life [14, 15]. Hemispheric asymmetry has been considered when examining brain activity and brain structure in patients with CP. Task-based fMRI studies have investigated the abnormal patterns of brain activation when performing one-side hand movement. Children with CP produced bilateral activity around the central sulcus, i.e., with more strongly ipsilesional activity in primary motor cortex and more activation in supplementary motor area (SMA) [16, 17], which indicated the alteration of brain activity asymmetry. In addition, abnormal diffusion parameters have been identified in corpus callosum (CC) of children with CP which correlate with hand function [17], suggesting structural impairment of interhemispheric connectivity. Also, lower fractional anisotropy (FA) within the posterior body and isthmus of the corpus callosum showed more significant correlation with motor dysfunction [18]. These previous studies highlight the importance of interhemispheric connectivity for motor and motor planning function in CP. However, the functional alteration of interhemispheric interactions and laterality of brain organization in dyskinetic CP during resting state are less known.

FMRI has been a powerful approach to noninvasively investigate the brain hemispheric asymmetry. Voxel-mirrored homotopic connectivity (VMHC) is an approach to examine the robust interhemispheric homotopic functional connectivity in the resting-state and provides insights regarding healthy development and disease process [19]. Previous studies have showed that sensorimotor cortex exhibits strong homotopic functional connectivity, while the homotopic connectivity in prefrontal, temporoparietal association areas is weaker [3]. In addition, lateralized networks of the brain associated with higher cognitive functions, such as left and right frontoparietal network, have also been identified using independent component analysis (ICA) [20, 21]. Resting state networks (RSNs), defining distinct modes of long-distance interactions, have been widely used to investigate the brain function, and the spatial lateralization of the intrinsic RSNs may reflect the functional organization in different states of the brain [22, 23]. Therefore, we hypothesized that the interhemispheric homotopic functional connectivity and the lateralization of intrinsic RSNs may be altered in DCP, and the interhemispheric interaction of the sensorimotor system in brain may be decreased, while the laterality of the RSNs associated with high cognitive function may be altered.

To test the hypothesis, VMHC and lateralization of the RSNs were calculated for children with DCP and healthy children. Group comparisons between groups were performed. Then, relationship between the interhemispheric interaction and motor-related clinical measurements in DCP was then examined.

## 2. Methods

### 2.1. Participants

A total of 24 children with DCP were recruited from Sichuan Rehabilitation Hospital (12 female, mean age: 8.5 years, age range: 3-18 years). Diagnosis for dyskinetic CP was made through standardized assessment by neurologists based on clinical features and MRI scanning [8]. All children with DCP in this study were dystonic. The inclusion criteria for the study were (1) diagnosis of DCP with predominant dyskinetic features; (2) age under 18 years old; (3) no history of trauma or brain operation, and no child was on medication. Besides, 20 healthy children without history of neurological disorder or brain injury (9 female, mean age: 8, age range: 4-13) were included in health control group (HC). All the participants are right-handed. This study was reviewed and approved by the Ethics Committee of the University of Electronic Science and Technology of China (UESTC). Parents of all participants gave written informed consent in accordance with the Declaration of Helsinki.

### 2.2. Clinical Measurement

Motor function of DCP subjects was assessed by the Gross Motor Function Classification System (GMFCS) [24]. GMFCS divides motor ability into five ordinal levels based on sitting, walking, and wheeled mobility, from level I indicating children with no disability for community mobility to level V indicating children totally dependent on assistance for mobility. Activities of daily living (ADL) [25] was also evaluated by the Assessment of Motor and Process Skills (AMPS) to access the self-care ability during everyday life. GMFCS and ADL scores for children with DCP were evaluated by physical therapists in Rehabilitation assessment department, Sichuan Rehabilitation Hospital.

### 2.3. Data Acquisition and Data Preprocessing

All participants were scanned using a 3T MRI Scanner (GE DISCOVERY MR750) at the MRI Research Center of UESTC. High-resolution T1-weighted images were obtained by a three-dimensional fast spoiled gradient echo sequence. The acquisition parameters are as follows: repetition time (TR) = 5.956ms, echo time (TE) = 1.964ms, flip angle (FA) = 9°, matrix= 256 × 256, field of view (FOV) = 25.6 × 25.6 cm^2^, and slice thickness = 1 mm; no gap, 152 slices. T2 images were also acquired using OAx T2 fluid attenuated inversion recovery (OAx T2 FLAIR) (TR= 8400ms, TE =150ms, FA = 111, matrix = 256 × 256, FOV = 25.6 × 25.6 cm^2^, and slice thickness = 4 mm). Resting state functional images were acquired using a single-shot, gradient-recalled echo planar imaging sequence (TR = 2000 ms, TE = 30 ms, FA = 90°, FOV = 24 × 24 cm^2^, slice thickness = 4 mm, and slice gap = 0.4mm). The children were instructed to keep with their eyes closed and remain still. All subjects underwent a 510s scanning to yield a total of 255 volumes.

Preprocessing of data was performed using SPM12 (Statistical Parametric Mapping, http://www.fil.ion.ucl.ac.uk/spm/). Data preprocessing comprised (1) discarding the first ten volumes from all the resting-state scans for magnetization equilibrium; (2) slice timing correction; (3) realigned: 3D motion detection and correction; (4) spatial normalization using T1 image and resample (3 mm × 3 mm × 3 mm); (5) spatial smoothing using a 6 mm full width at half maximum Gaussian kernel. Children with head movement greater than 2 mm in any direction and head rotation greater than 2 degree were excluded. Comparison was performed for the individual mean frame-wise displacement (FD) calculated by averaging the relative displacement from every time point for each subject (Power et al., 2012), and no difference was found between the groups (two sample t-test, P=0.4291). In addition, nuisance signals, including six head-motion parameters, WM signal, and CSF signal according to individually estimated anatomical masks, were regressed out.

### 2.4. Voxel-Mirrored Homotopic Connectivity Computation

Before VMHC calculation, linear trends and a phase-insensitive filtering (0.01-0.08Hz) were applied to reduce the effects of low frequency drift and high frequency physiological noise. VMHC was defined as the Pearson correlation between the time series of each pair of symmetric interhemispheric voxels. To acquire group-specific symmetrical template, we averaged normalized T1 images of the 30 subjects (15 DCP and 15 HC) to create a mean normalized T1 image, and its left-right mirror version was applied to make the template. Then, all subjects' functional images were normalized to standard space using the symmetrical template. Correlation coefficients between homotopic voxels were then Fisher Z-transformed and weighted by the voxel-wise gray matter tissue probability values to obtain each voxel's Z-value [19]. VMHC for two groups was calculated using DPABI (http://rfmri.org/dpabi) [26].

### 2.5. Lateralization of RSNs

#### 2.5.1. ICA Decomposition and RSNs Extraction

Spatial group ICA for DCP and HC was conducted in GIFT version 4.0a [27] (http://mialab.mrn.org/software/gift/). ICA decomposition produced independence components (ICs) denoting a group of brain regions with unique patterns. Principal component analysis (PCA) was adopted for the reduction of data dimensionality. The number of the ICs was determined by the minimum description length (MDL) [28], and ICs estimation was repeated 20 times in ICASSO (http://research.ics.tkk.fi/ica/icasso). Then, the ICs of each subject were backconstructed using the dual-regression approach. The grey mask used in this process was created by the T1 segmented images of two groups. The RSNs were identified based on the average power spectra and spatial pattern of the components [29, 30]. Thus, the time-courses and spatial maps were acquired for each participant, and the participant-specific maps were converted to Z-scores.

#### 2.5.2. Lateralization of RSNs Calculation

Lateralization of RSNs (*B*_*V*_ values) was calculated by the difference of homotopic voxels for the IC values between the left and right cerebral hemispheres [22, 23]. Previously, standard MNI template was used to make a symmetrized template using the warping step, and ICs of all subjects were normalized to this symmetrized template. Then, *B*_*V*_ was calculated using the following formula (1). In this formula, *R* and *L* separately represented the Z-scores for the homotopic voxels of the left and right hemispheres. The voxel-wise laterality map can be produced with positive difference on the right side of the brain when *R* > *L* and positive difference on the left side of the brain when *L* > *R*. One sample t-test for the normalized ICs was used to make a mask to limit *B*_*V*_ values. Finally, the *B*_*V*_ values can be displayed in each RSN.

(1) $$\begin{array}{c} Bv=\left\{\begin{array}{cc} \left(Rv-Lv\right) & if\;\;Rv>Lv\\ \left(Lv-Rv\right) & if\;\;Lv>Rv\\ 0 & otherwise \end{array}\right. \end{array}$$

#### 2.5.3. Laterality Cofactor

The laterality cofactor (LCF) was defined as a metric of global laterality for a given RSN. As can be seen in formula (2), the LCF was obtained by taking the difference between the sum of all intensities of laterality on the right and left hemispheres regarding to the sum of all intensities across the brain.

(2) $$\begin{array}{c} LCF=\frac{S_{r}-S_{l}}{S_{r}+S_{l}} \end{array}$$

*S*_*l*_ and *S*_*r*_ represent the sum of all intensities for one RSN on the left and right hemisphere.

### 2.6. Relationship between the Interhemispheric Organization and the Clinical Measurements

Correlations were calculated between the interhemispheric functional organization metrics, i.e., VMHC and *B*_*V*_, and the clinical measurements of CP. The regions showing significant difference for VMHC and *B*_*V*_ between groups were chosen as the regions of interest (ROIs) (Tables 2 and 3), and the coordinates of the ROIs were extracted. Then, the mean Z-scores of VMHC and the mean *B*_*V*_ values within the ROI were used for the correlation calculation with the GMFCS and ADL scores.

### 2.7. Statistical Test

Before statistical analyses, normality of the data distribution was tested using the Lilliefors' test, including the age, ADL scores, Z-maps of VMHC, and RSNs for each voxel. As for VMHC, two-sample t-test was performed to examine the difference between DCP and HC groups, and marked regional differences were demonstrated (P<0.001). For *B*_*V*_, the group-level *B*_*V*_ map for each RSNs was obtained using one-sample t-test including all the subjects in two groups. The t-value maps for *B*_*V*_ applied a mask generated from the one-sample t-test (threshold P<0.001) of Z-maps for each RSN across subjects. Then, two-sample t-test with statistical significance level P < 0.005 was conducted to detect the group difference between DCP and HC for *B*_*V*_.

## 3. Results

### 3.1. Demographic Information

Cerebral abnormalities of children with DCP were first evaluated by two radiologists according to the high-resolution T1 (3D FSPGR) and T2 (OAx T2 FLAIR) images. Among 24 children with DCP, 6 children have cortical gray matter lesions and 3 children with slight basal ganglia abnormality, mainly located in bilateral putamen. Nine children have some white mater lesions, predominately consisting of periventricular leukomalacia. In the following analysis, children with obvious cortical lesion (6 children) and large head motion (3 children) during MRI scanning were excluded. In addition, 5 healthy children were excluded because of the large head motion during MRI scanning. Finally, 15 children with DCP (6 female, mean age: 9.6 years, age range: 3-16 years) and 15 healthy children (7 female, mean age: 9.5, age range: 4-13) meeting the criteria were included in the following VMHC and ICA processing. No significant difference was found for age and gender between two groups (two-sample t-test for age, P=0.98; chi-square test for gender, P=0.713). Demographic and clinical data of the sample were shown in Table 1.

### 3.2. Group Comparison of VMHC

**Figure 1:**
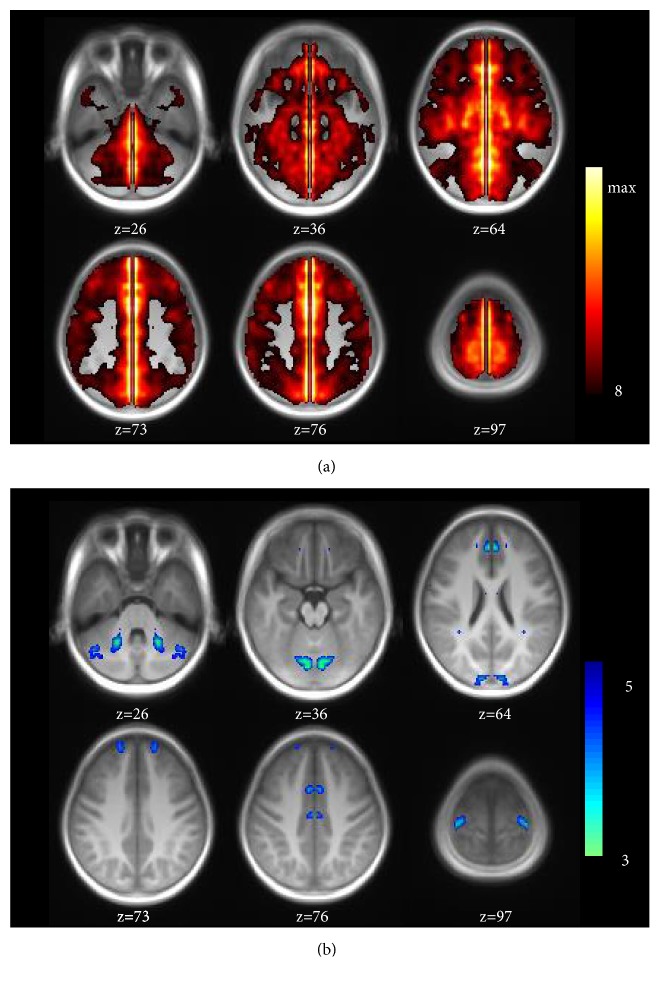
(a) Group-level of VMHC maps resulting from the one sample t-test for all subjects in two groups (P<0.0001). (b) Group comparison result of the VMHC by two sample t-test overlaying on the symmetrical template (P < 0.001).

The group comparison for VMHC was conducted between DCP and HC (Figure 1).

Compared with HC, DCP observed decreased interhemispheric functional connectivity mainly in motor and pre-motor related areas, including cerebellum, precentral, SMA, anterior cingulate (ACC), middle cingulate (MCC), and bits of frontal and calcarine areas. The regions with significant difference in group comparison were shown in Table 2.

### 3.3. RSNs Identification

**Figure 2:**
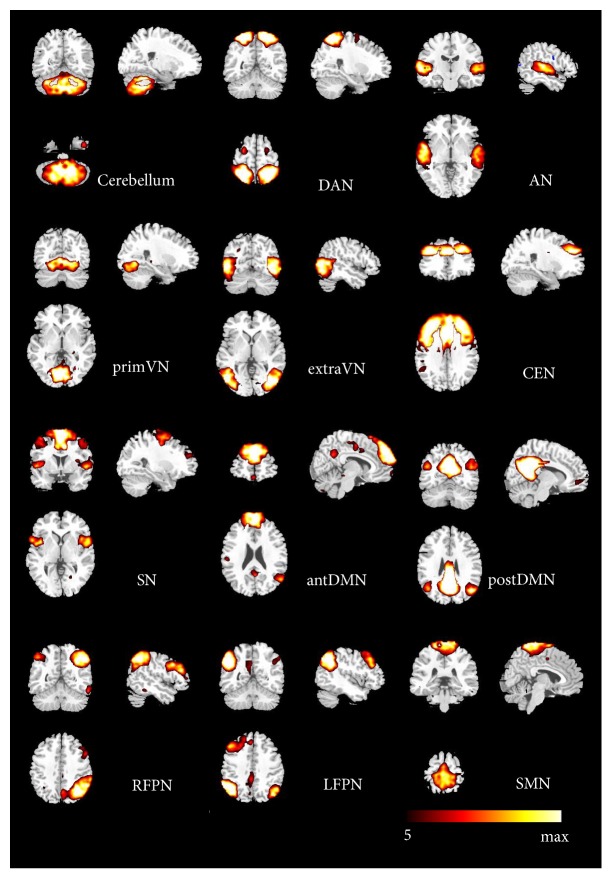
The spatial maps of 12 RSNs identified according to the group ICA.

12 components were selected as the resting-state relevant networks from the group ICA [31, 32]. No clusters in each RSN fell within the lesion of any of the children. The spatial maps of the 12 RSNs are illustrated in Figure 2. These networks are labeled as follows: (1) cerebellum network: the spatial patterns encompassing the cerebellum posterior lobe and decline; (2) DAN: dorsal attention network mainly including the bilateral intraparietal sulcus, frontal eye field and middle temporal lobe; (3) AN: the auditory network primarily involving the middle temporal gyrus, superior temporal gyrus corresponding to the auditory system; (4) primVN: the primary visual network consisting of the calcarine, cuneus, and lateral lingual gyrus; (5) extraVN: the extrastriate visual network encompassing the bilateral fusiform gyrus, middle temporal, and middle occipital areas; (6) CEN: the central executive network showing spatial patterns comprising the middle and superior frontal cortices, anterior cingulate, and paracingulate gyri; (7) SN: the salience network mainly consisting of dorsal anterior cingulate (dACC), orbital frontoinsular cortices, and part of prefrontal areas; (8) antDMN: the anterior part of default mode network (DMN) including the superior frontal gyrus and middle frontal gyrus; (9) postDMN: the posterior part of DMN, involving the precuneus, the posterior cingulate cortex (PCC), and bilateral angular gyrus; (10) LFPN: the left lateral frontoparietal network involving the left middle frontal gyrus, inferior parietal lobule, superior parietal lobule, and angular gyrus; (11) RFPN: the right lateral frontoparietal network showing the mirrored spatial patterns with LFPN. LFPN and RFPN were the only maps strongly lateralized and left-right mirrors of each other; (12) SMN: sensorimotor network including the supplementary motor area, the paracentral lobule, and pre- and postcentral gyrus.

### 3.4. Group Comparisons of *B*_*V*_ within RSNs

**Figure 3:**
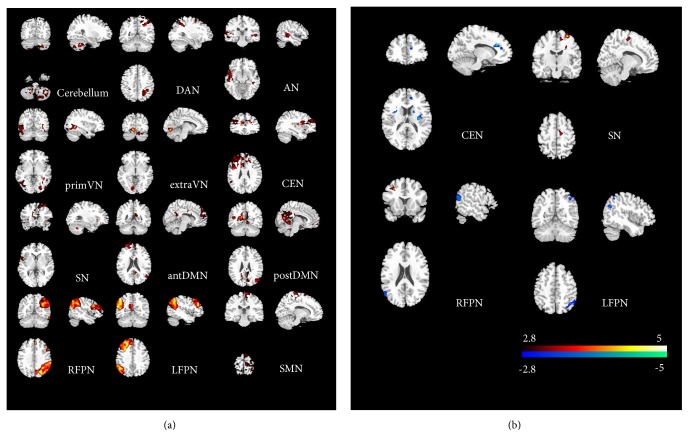
(a) Group-level of Bv maps resulting from the one sample t-test for all subjects in two groups (P<0.001). (b) Group comparison of Bv maps resulting from the two-sample t-test between groups (P<0.005).

For the identified 12 RSNs, their *B*_*V*_ maps were calculated for each child. The group level maps resulting from the one-sample t-test of all children in two groups were shown in Figure 3(a). *B*_*V*_ maps between DCP and HC were compared by two-sample t-test (threshold at P<0.005) within each RSN (Figure 3(b)). Comparing to HC, DCP revealed significant weaken *B*_*V*_ in ACC and insula for CEN. Moreover, altered *B*_*V*_ was found in LFPN and RFPN in DCP. In addition, DCP also showed increased *B*_*V*_ in SMA and precentral areas of SN. The regions with significant difference between groups were shown in Table 3.

### 3.5. Laterality Cofactors

**Figure 4:**
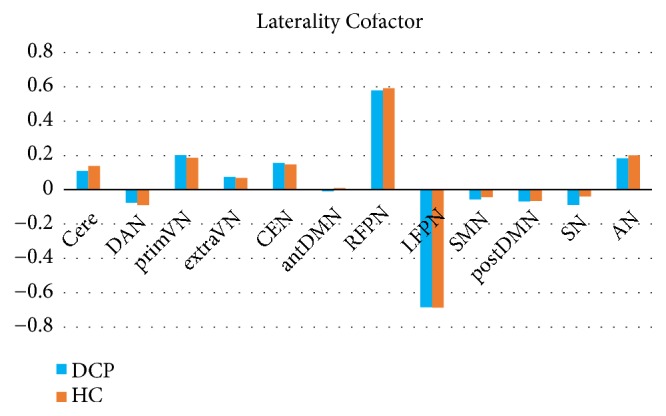
Global LCF within 12 RSNs in DCP and HC.

Within the two groups, the global LCF of 12 RSN was shown in Figure 4. Generally, the laterality cofactors in both groups with DCP and healthy children are much smaller than those in adult groups in previous studies [22, 23]. According to the previous criteria [22], we found the LCFs were lateralized in RFPN and LFPN in both groups (LFCs larger than 0.2). The laterality of the remaining of 10 RSNs was not significant. However, no significant difference was found between the two groups for laterality cofactors in all RSNs.

### 3.6. Relationship between VMHC/*B*_*V*_ and Clinical Measurement

**Figure 5:**
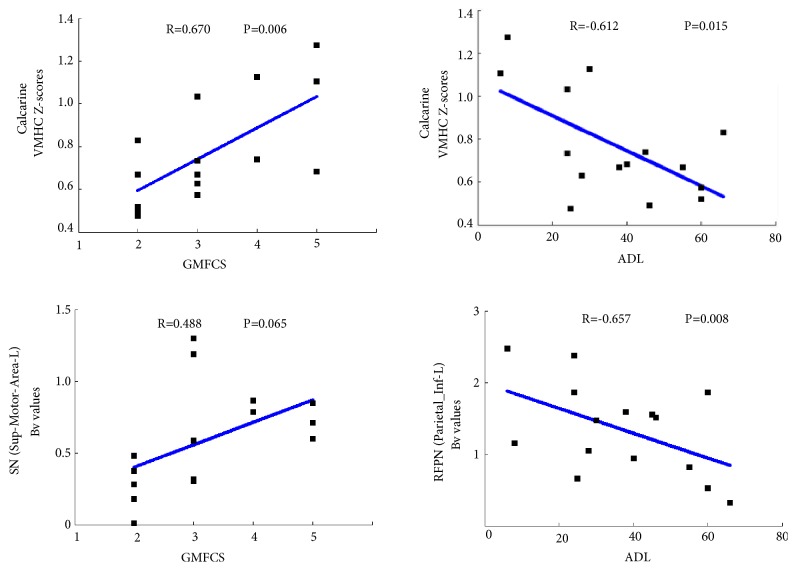
Relationship between VMHC / Bv lateralization and the clinical measurements in DCP.

Correlations were performed between *B*_*V*_/VMHC scores in CP group and the clinical measurement. The results were shown in Figure 5. Inferior parietal lobule in RFPN showed negative correlation with ADL scores, and SMA showed positive correlation with GMFCS (P<0.05). Moreover, significant correlations were found between the VMHC in calcarine and clinical scores, including the negative correlation with GMFCS and positive correlation with ADL scores (P<0.05).

## 4. Discussion

The lateralization of human brain function is associated with function organization and functional specialization. This study investigated the interhemispheric functional organization of the brain in DCP and HC group applying the interhemispheric connectivity and lateralization of intrinsic networks based on the resting state fMRI. First, comparing with healthy controls, DCP group demonstrated an altered voxel-mirrored interhemispheric connectivity mainly in motor-related areas. Furthermore, the RSNs laterality analysis showed abnormal lateralization in cognitive-related networks for children with DCP. These findings supposed that altered brain organization widely existed in children with DCP, which may extend our understanding for CP dysfunction.

Cerebral palsy is generally considered as motor-related disease, and many fMRI studies have focus on the organization of sensory and motor pathways between sensorimotor cortex and the affected hands [16, 33]. Increased bilateral primary sensorimotor cortices and ipsilateral SMA were found during affected hand movement [17, 34]. Bilateral activation may result from the existence of lack of inhibition mediated transcallosally, mirror movements, or ipsilateral projections, which suggests the abnormal brain asymmetry in CP. In VMHC analysis of this study, the altered interhemispheric connectivity is mainly located in motor and motor control related areas, including the cerebellum, precentral, MCC, and the frontal, ACC, and visual area, which were highly consistent with the results of the previous seed-based fMRI connectivity study that the motor cortical connectivity was diminished mainly within the bilateral somatosensory cortex, paracentral lobule, cingulate motor area, and visual cortex in CP group [18]. The impairment of sensorimotor integration in hemiplegic cerebral palsy, including the contralateral somatosensory representation and the transhemispherically reorganization for primary motor cortex in CP group, may suggest an interhemispheric dissociation for the sensorimotor inputs and output. Studies based on diffusion tensor imaging showed reduced WM integrity in corpus callosum (CC) for children with CP, and the WM integrity was correlated with hand functions, demonstrating the impaired interhemispheric structural connectivity in CP [17]. The decreased voxel-mirrored interhemispheric connectivity supposed that the abnormal sensorimotor reorganization in CP may be related with the altered interhemispheric connectivity of sensorimotor cortex. In the present study, we also found interhemispheric connectivity deficits involving frontal and ACC areas, which were important areas related to executive and motor-control function. Further, ACC plays a central role in the executive function network of brain, being highly connected with the prefrontal cortex, premotor, and supplementary motor areas and parietal cortex [35, 36]. Besides, the altered interaction between hemispheres in the visual areas may be associated with the visuomotor function impairment in children with CP.

Besides motor function impairment, children with CP tend to be associated with many cognitive impairments such as attention, learning, and memory disability [8, 37]. In RSNs lateralization analysis, the altered lateralization was mainly located at the cognitive-related networks, including CEN, SN, and frontoparietal network (FPN). CEN and FPN are two important cognitively demanding networks. CEN is well engaged in cognitively demanding tasks, manipulating information about the external environment [38], and ACC and insula are two important nodes in CEN network. The FPNs are associated with attention, cognitive performance, and control processes, and altered functional connectivity in FPNs has been found in previous study in children with CP [30, 39]. As two distinguished asymmetric networks, LFPN has been mentioned to correspond well to cognition–language paradigms, while RFPN is a network related to visual-spatial attention and somesthesis perception [40]. Evidence points to children with CP commonly experienced abnormal attention and executive functions deficits [41, 42], which may be related with the altered laterality of FPNs. In addition, the increased lateralization of precentral cortex and SMA may be related with the decreased voxel-mirrored interhemispheric connectivity in the same area, commonly resulting in the impaired organization of sensorimotor function.

There are several limitations which should be mentioned here. Firstly, there are a variety of brain lesions in CP group, and potential structural effect may exist on functional analysis. Therefore, we adopted strict inclusion criteria to exclude the children with obvious brain lesions to eliminate the structure effect. The final cohort involved 3 children with slight basal ganglia lesions and no child with cortical grey matter abnormality. In addition, there were only GMFCS and ADL measurements in this study, and no specificity in motion and cognitive scores was applied. Moreover, the sample size is relatively small in this study, as the children with cortical lesions and large head motion during MRI scanning were excluded. Future studies should include larger sample size to determine the mechanism underlying the abnormal brain laterality found in the current study. Finally, ICA as an unsupervised method applied to identify the RSNs in the study still presented a challenge in including physiological information in current algorithm framework.

## 5. Conclusion

In conclusion, interhemispheric connectivity and lateralization of functional networks are important organization of the human brain, which are intensively distorted by cerebral palsy. In this study, the VMHC analysis showed decreased homotopic interhemispheric functional connectivity in motor and motor-control related regions in DCP group. Altered lateralization of intrinsic resting-state networks was found in cognitive-related networks. These findings provide the view from the interhemispheric functional organization and may demonstrate a limited compensatory potential for understanding the underlying pathological mechanism of CP.

## Figures and Tables

**Table 1:** Demographic and clinical data of the sample.

	DCP	HC	P-value
*Number of participants (N)*	24	20	
Age (mean years±std)	8.5±4.6	9±2.2	0.864
Gender	12 M, 12 F	12 M, 8 F	0.636
GMFCS	II:5, III:9, IV:9, V:3		
ADL (mean scores±std)	38.44±18		
*Neuroimage finding (N)*			
White matter lesion	9		
Cortical gray matter abnormality	6		
Basal ganglia/Thalamus abnormality	3		
Normal (N)	8		

M: male, F: female, std: standard deviation, and N: number of participants

P value: comparisons for age and gender between two groups: the variables; gender was analyzed using chi-square test, while the age was analyzed using two-sample t-test.

**Table 2:** Significant difference of VMHC in two-sample t-test between DCP and HC (P<0.001).

AAL Regions	MNI coordinates			Peak T-value	Cluster voxels
	X	Y	Z		
Cerebelum-Crus1-R	18	-72	-27	-6.6709	64
Cerebelum-6-R	9	-78	-20	-4.4921	63
Cerebelum-Crus1-L	-18	-72	-27	-6.6709	92
Cerebelum-6-L	9	-78	-20	-4.4921	49
Calcarine-R	6	-93	3	-7.1631	44
Calcarine-L	-6	-93	3	-7.1631	65
Cingulum-Ant-R	3	42	24	-6.7165	36
Cingulum-Mid-L	-3	12	39	-4.7916	25
Frontal-Sup-R	18	57	36	-4.9683	29
Precentral-L	-33	-24	69	-5.8863	48
Precentral-R	33	-24	69	-5.8863	58
Cingulum-Mid-R	3	12	39	-4.7916	28
Supp-Motor-Area-R	12	-24	46	-4.6234	23

**Table 3:** Significant difference of Bv in two-sample t-test between DCP and HC (P<0.005).

Networks	AAL Regions	MNI coordinates			Peak T-value	Cluster voxels
		X	Y	Z		
CEN	Cingulum-Ant-R	15	27	27	-6.6985	66
	Insula-R	33	-18	15	-4.6162	29
LFPN	Temporal-Mid-R	57	-66	12	-4.4848	72
	Frontal-Mid-R	30	15	39	5.0786	46
RFPN	Angular-L	-42	-66	27	4.3089	26
	Parietal-Inf-L	-54	-60	45	-4.0177	55
SN	Supp-Motor-Area-L	-12	-18	57	3.9542	35
	Precentral-L	-30	-15	63	4.5851	24

## Data Availability

The data used to support the findings of this study are available from the corresponding author upon request.
